# Inhibitory control and visuo-spatial reversibility in Piaget's seminal number conservation task: a high-density ERP study

**DOI:** 10.3389/fnhum.2013.00920

**Published:** 2013-12-27

**Authors:** Grégoire Borst, Grégory Simon, Julie Vidal, Olivier Houdé

**Affiliations:** ^1^Laboratory for the Psychology of Child Development and Education, Psychology, CNRS Unit 3521, Sorbonne Paris Cité, University Paris Descartes, Université Caen Basse NormandieParis, France; ^2^Institut Universitaire de FranceParis, France

**Keywords:** number conservation, ERP, inhibitory control, reversibility, mental imagery

## Abstract

The present high-density event-related potential (ERP) study on 13 adults aimed to determine whether number conservation relies on the ability to inhibit the overlearned length-equals-number strategy and then imagine the shortening of the row that was lengthened. Participants performed the number-conservation task and, after the EEG session, the mental imagery task. In the number-conservation task, first two rows with the same number of tokens and the same length were presented on a computer screen (COV condition) and then, the tokens in one of the two rows were spread apart (INT condition). Participants were instructed to determine whether the two rows had an identical number of tokens. In the mental imagery task, two rows with different lengths but the same number of tokens were presented and participants were instructed to imagine the tokens in the longer row aligning with the tokens in the shorter row. In the number-conservation task, we found that the amplitudes of the centro-parietal N2 and fronto-central P3 were higher in the INT than in the COV conditions. In addition, the differences in response times between the two conditions were correlated with the differences in the amplitudes of the fronto-central P3. In light of previous results reported on the number-conservation task in adults, the present results suggest that inhibition might be necessary to succeed the number-conservation task in adults even when the transformation of the length of one of the row is displayed. Finally, we also reported correlations between the speed at which participants could imagine the shortening of one of the row in the mental imagery task, the speed at which participants could determine that the two rows had the same number of tokens after the tokens in one of the row were spread apart and the latency of the late positive parietal component in the number-conservation task. Therefore, performing the number-conservation task might involve mental transformation processes in adults.

## Introduction

New imaging technologies, such as high-density electroencephalogram (EEG), may help us answer some of the oldest and deepest questions regarding what logicomathematical cognition is and how it works. According to Piaget's (1952) seminal work, successes in conservation tasks reveal a fundamental qualitative change in the logicomathematical intelligence of children: At ~7 years old, children are not only able to represent static states (i.e., the preoperational stage) but also mentally manipulate internal representations (i.e., the concrete operations stage). All conservation tasks require children to grasp the idea that the transformation of one dimension of an object (or a display of objects) does not affect the other dimensions of that object. For instance, in the seminal number-conservation task, two rows with an identical number of objects are initially presented with the objects in the two rows yielding a one-to-one correspondence. When the children acknowledged that the two rows possess the identical number of objects (i.e., initial equivalence), one of the rows is transformed in length but not in number (e.g., the objects are spread apart). Children are again asked whether the two rows have an identical number of objects. Before the age of 7, children erroneously think that the longer row contains more objects regardless of the number of objects in each row. To understand that the transformation of the length of the row does not affect the number of objects in that row, children must grasp that operations (such as transformation) are reversible, e.g., the movement from A to B (i.e., the lengthening of the row) can be eliminated by the movement from B to A (i.e., the shortening of the row).

However, studies have provided evidence that young children have some knowledge of numbers (e.g., Mehler and Bever, 1967; Gelman, 1972; Antell and Keating, 1983; van Loosbroek and Smitsman, 1990; Wynn, 1992). In particular, neonates appear to have the ability to perceive numerical invariance in situations similar to those in Piaget's number-conservation task, in which length and number conflict (Antell and Keating, 1983). The discrepancy between the age at which children succeed at Piaget's number-conservation task and the early numerical abilities reported in younger children can be partially explained by the strategy selected by children to solve a problem (Siegler, 1987). According to the information-processing theory of cognitive development [see Siegler (1998)], children are limited by the amount of information they can process. Children use various strategies to overcome this information-processing constraint. When an identical problem is encountered, children will progressively shift from procedural to heuristic (i.e., a quick and most of the time efficient strategy) strategies (Siegler, 1996). Hence, at any age, one possesses several competing strategies for solving a problem (e.g., Shrager and Siegler, 1998; Siegler, 2007). Moreover, the selection of the appropriate strategy relies on the ability to inhibit irrelevant or misleading strategies, particularly in interference tasks such as number-conservation (Houdé, 2000, 2007).

Several studies converge in showing that the inhibition of the length-equals-number heuristic (i.e., longer alignments contain more objects than shorter ones) is critical to succeed at Piaget's number-conservation task in children (Houdé and Guichart, 2001; Houdé et al., 2011; Poirel et al., 2012) and adults (Daurignac et al., 2006; Leroux et al., 2006, 2009; Joliot et al., 2009). A fronto-parietal network, including regions involved in inhibitory control, such as the right inferior frontal gyrus/insula, is activated in children who succeed at the number-conservation task but not in children who fail (Houdé et al., 2011). A follow-up study revealed that the level of activation within the right inferior frontal gyrus/insula was selectively related to the inhibitory control efficiency of children assessed with the Animal Stroop task (Wright et al., 2003; Poirel et al., 2012).

In adults, event-related potential (ERP) studies revealed modulations of the amplitudes of the N2 and P3 components in adaptations of Piaget's number-conservation task (Leroux et al., 2006, 2009; Joliot et al., 2009). The amplitudes of the N2 and P3 components increased in items whose length and number conflict (e.g., the longer and shorter rows contain an identical number of objects) compared to items whose length and number co-vary (e.g., the longer row contains more objects than the shorter row). The N2 and P3 components reflect the inhibition of motor responses, such as in the Go/No-Go tasks (e.g., Falkenstein et al., 1999; Kok, 1999; Mathalon et al., 2003), and the more general processes of monitoring conflicts and dominant responses inhibition (e.g., Kok, 1999; Nieuwenhuis et al., 2003; Anokhin et al., 2004). Thus, adults and children must detect the presence of a conflict between a length and number and then inhibit the length-equals-number heuristic to determine that the two rows have similar numbers of objects when the length and number interfere.

However, little is known of the strategy that is activated when the length-equals-number heuristic is efficiently inhibited. In addition, the need for adults to inhibit the length-equals-number heuristic when object spreading is presented visually, as in Piaget's seminal number-conservation task, is questionable. Therefore, the aim of the present behavioral and ERP study was two-fold: (1) To determine whether adults inhibit the length-equals-number heuristic even when the transformation of the length of one row is visually displayed after the acknowledgment of the initial equivalence and (2) to better characterize the solving strategy employed by adults in the number-conservation task. Providing information on the strategy used by adults is critical to understand the mechanism by which children acquire number conservation and more generally the concept of conservation. Two divergent views of these mechanisms have been proposed in developmental psychology (e.g., Piaget, 1952, 1967; Bruner, 1964, 1966). According to Piaget (1952), there are three basic operation accounts for children's understanding of the conservation problem: Identity (e.g., nothing was added or subtracted during the transformation of the length of the row), compensation (e.g., one row is longer, but the gap between the objects is narrower in the other row), and reversibility (e.g., the movement from A to B can be eliminated by the movement from B to A). The structure of all three operations is logico-propositional. However, Bruner (1964, 1966) argues that the acquisition of conservation is rooted in learning to progressively disentangle the principle of identity from the perceptual conflicts that occur in all conservation problems, such as the conflict between length and number in the number-conservation task. Children are assumed to learn to conserve discontinuous and continuous quantities through the manipulation of iconic representations (i.e., mental images) and language. Critically, Bruner claims that reversibility is not critical for solving conservation problems and could be akin to a manipulation of mental images.

In the present study, we recorded high-density ERPs from young adults performing an adaptation of Piaget's number-conservation task to determine whether the inhibition and reversibility of operations are necessary for number conservation. In each trial, first two rows with the same number of blue tokens and the same length were presented on a computer screen Thus, in this condition, the length co-varied with the number of tokens—i.e., the two rows had the same length and the same number of tokens (hereafter we referred to this condition as the COV condition). The participants were instructed to determine whether the two rows contained an identical number of tokens. Then, the tokens in one of the two rows were spread apart, and the participants were again asked whether the two rows had an identical number of tokens. Thus, in this condition, the length of the rows interfered with the number of tokens—i.e., the two rows had different lengths but the same number of tokens (hereafter we referred to this condition as the INT condition). We varied both the number of tokens in each row (in the COV and the INT conditions) and the difference in the distance between the tokens in the two rows after the tokens in one row were spread apart (in the INT condition). This procedure mirrored the three critical phases of Piaget's conservation task: initial equivalence (COV condition), transformation and conservation question (INT condition). We reasoned that if adults must inhibit the length-equals-number heuristic to perform Piaget's number-conservation, even when the transformation of the row length is presented visually, then (a) the response times (RTs) should be higher in the INT condition than the COV condition and (b) the amplitude of the N2 and P3 components should be higher in the INT condition than the COV condition. In addition, if these ERP components reflect the ability to inhibit the length-equals-number heuristic in the number-conservation task, then the difference in the RTs between the INT and COV conditions should be correlated with the difference in the amplitudes of the ERP components between the INT and COV conditions. Note that larger differences in the RTs and ERP amplitudes between the two types of items would reflect a poor inhibitory control efficiency, as in the Stroop color word task (e.g., Stroop, 1935; MacLeod, 1991).

To determine whether the reversibility of operations was (a) necessary to perform the number-conservation task and (b) consisted of imagining a shortening of the row that was lengthened, participants performed a mental imagery task after completing Piaget's number-conservation task. In this task, the stimuli presented were similar (i.e., same numerosity and differences in distance between the tokens in the two rows) to the ones in the INT condition of the number-conservation task except that no transformation of the length of one of the row was apparent. In each trial, the participants were simply instructed to mentally imagine the tokens in the longer row aligning with the tokens in the shorter row and to press a button when they finished imagining this shortening. Mental imagery studies consistently reported linear increases of RTs with increasing physical transformation of the object, for instance, increasing angular disparity between the objects in a mental rotation task (e.g., Shepard and Metzler, 1971; Borst et al., 2011). Many researchers have interpreted these effects as showing that mental images are transformed in a manner that emulates the corresponding physical transformation (e.g., Kosslyn et al., 2006). Critically, the speed (i.e., efficiency) to perform such mental transformation is reflected by the slopes of the best-fitting line between the RTs and physical variable manipulated, such as the angular disparity between the objects in the mental rotation task (e.g., Borst and Kosslyn, 2008, 2010; Borst et al., 2011). In addition, ERP studies revealed that mental transformation processes, such as mental rotation, are indexed by a positivity 300–700 ms at the parietal channels after stimulus presentation (e.g., Heil, 2002; Schendan and Lucia, 2009; Beste et al., 2010). We reasoned that if adults rely on a reversibility of operations akin to eliminating the lengthening of one row by imagining the reverse transformation, then (a) the RTs should increase linearly with the increasing differences in the distance between the tokens in the two rows in the mental imagery task and the INT condition of the number-conservation task, (b) the speed of mentally shortening the row, as reflected by the slopes of the best-fitting lines between the RTs and differences in the distance between the tokens in the two rows, should be related in the two tasks, and (c) the slopes of the best-fitting lines should be correlated with a late positive parietal component.

## Methods

### Participants

Thirteen healthy adults (8 females, 5 males), 20–31 years old (mean age of 24.9 ± 0.9 years) volunteered to participate in this experiment. All participants were undergraduate university students living in the same urban environment (Paris, France). All participants had normal or corrected-to-normal vision. All participants provided written informed consent and were tested in accordance with national and international norms governing the use of human research participants.

### Materials and procedure

The participants were comfortably seated ~80 cm in front of a 17-inch LCD monitor linked to a PC computer equipped with E-prime 2.0 Professional software (Psychology Software Tolls, http://www.pstnet.com). The stimuli were displayed over a black background on an LCD computer screen with a visual angle of 15°. During the EEG session, the participants performed the number-conservation task. The EEG session lasted ~1 h: 30 min for the preparation and installation of the participants and 30 min for completing the number-conservation task. After the EEG session, all participants performed the mental imagery task on the identical computer. The order of the task was not counterbalanced across participants to prevent the induction of a mental imagery strategy that otherwise would not have been used spontaneously in the number-conservation task.

#### Number-conservation task

The stimuli consisted of two rows of blue tokens (12 mm in diameter) within a large white rectangle (21 cm long, 17 cm wide) divided in two by a horizontal black line. One row was displayed above this line and the other row below the line. The two rows contained either the identical (5, 6, 7, or 8 tokens in both rows) or a different number of tokens (5 vs. 4, 6 vs. 5, 7 vs. 6, or 8 vs. 7 tokens in each row).

**Figure 1:**
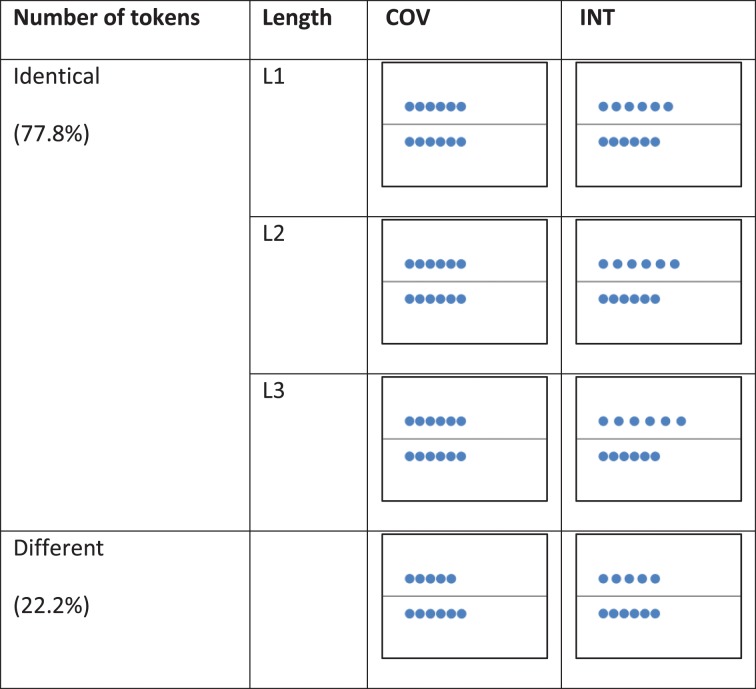
**Examples of stimuli presented in the COV and the INT conditions of the number-conservation task**.

**Figure 2:**
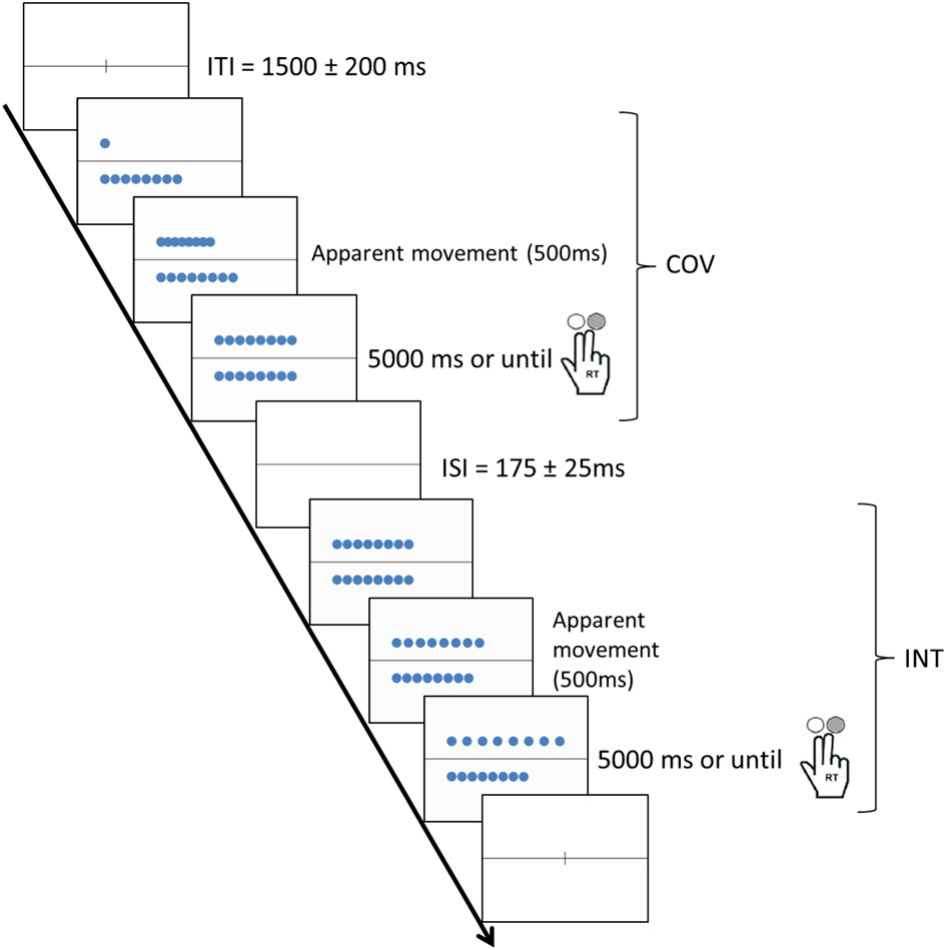
**Procedure used in the number-conservation task when the COV stimulus was presented first**. The COV and INT stimuli both involved an apparent movement (500 ms) that started at the stimulus onset. Note that all participants provided their responses by pressing the “yes” or “no” buttons on the response box with the forefinger and middle finger of their right hand and that COV and INT stimuli remained on the screen until participants provided an answer (or for 5000 ms if participant did not press one of the two response-buttons).

Each trial involved performing a COV stimulus and an INT stimulus. In the COV stimuli, the number of tokens and length of the rows co-varied, whereas in the INT stimuli, the number of objects and lengths of the rows conflicted, i.e., the two rows contained an identical number of tokens but were of different lengths or the two rows were of the same length but contained different number of tokens (see Figure 1). The COV stimuli were presented initially followed by the INT stimuli, except in 11.1% of the trials (i.e., the distractor trials) to prevent habituation in the participants. The COV and INT stimuli both involved an apparent movement (500 ms) that started at the stimulus onset: In the COV stimuli, a row of tokens was displayed above or below the line, and the tokens of the second row, first superimposed on the left of the screen, were then progressively spread apart to align in a one-to-one correspondence with the tokens of the first row displayed. In the INT stimuli, the tokens of one of the two rows were spread apart with a 3-, 5-, or 7-mm increase in the initial distance between the tokens of the row displayed in the COV stimulus presented previously. Thus, the initial position of the tokens in the INT stimulus was always the final position of the tokens in the preceding COV stimulus (see Figure 2).

At the beginning of each trial, a fixation cross was superimposed on the horizontal line for 1300–1700 ms (1500 ± 200 ms). The two rows of tokens remained on the screen until the participants provided an answer or up to 5000 ms. Between the COV and the INT stimuli, the horizontal black line was displayed for 150, 175, or 200 ms. In each trial, the participants were asked to separately judge the numerical equivalence of the two rows of tokens in the COV and INT stimuli. The participants provided their responses by pressing the “yes” or “no” buttons in the response box with the forefinger and middle finger of their right hand, respectively. In 77.8% of the trials, the two rows possessed the same number of tokens. We randomized the presentation of trials with rows containing identical numbers of tokens and those containing different numbers. The participants performed 144 trials (i.e., pairs of COV-INT stimuli). For each item, we recorded the button pressed and RTs, i.e., the time between the onset of the stimulus and the button press. The participants performed 4 blocks of 36 trials with short breaks between each block to allow the participants to rest their eyes.

#### Mental imagery task

The stimuli were similar to the INT stimuli except with no apparent movement. Only the INT stimuli with an identical number of tokens in both rows were used. The distance between the tokens in the longer row was increased by 3, 5, or 7 mm from the one in the shorter row, regardless of the number of tokens in that row (5, 6, 7, or 8 tokens). The longer row was displayed above or below the horizontal line. In each trial, after the presentation of a fixation cross superimposed on the horizontal line dividing the screen (500 ± 100 ms), one stimulus was displayed for 5000 ms or until the participants provided a response. The participants were instructed to visualize the displacement of the tokens in the longer row until the tokens were aligned with those in the shorter row in a one-to-one correspondence. The participants pressed the space bar when they had mentally shortened the longer row. RTs were recorded from the onset of the stimulus to the space bar press. The participants performed 6 blocks of 48 trials.

### Psychophysiological recording and analysis

The electroencephalogram (EEG) was recorded from 256-channel HydroCel Geodesic Sensor Net (Electrical Geodesics Inc., Eugene, Oregon, USA) containing electrodes imbedded in small sponges soaked in a potassium chloride saline solution. Continuous EEG was acquired through DC amplifier (Net Amps 300 1.0.1, EGI) and digitized at a sampling rate of 250 Hz. A common reference at the vertex was used during acquisition and electrode impedances were kept below 100 kΩ. Eye-blinks and eye-movements were monitored via pairs of channels included in the net and covering face area. Processing stages described below were performed using the EGI NetStation 4.5 Waveform Tools.

The EEG was filtered offline with a first-order high-pass at 0.01 Hz and 40-Hz low-pass and then segmented from −100 to +1000 ms relative to the onset of each stimulus. Segments with a correct response (*RT*s > 200 ms) were binned into two categories: COV or INT. Voltages exceeding ± 200 and <0.5 μV (to exclude the flat channels), eye blinks exceeding ± 200 μV and eye movements exceeding ± 80 μV were rejected. Note that the number of segments rejected due to eye movements was identical in the COV (*M* = 4.03 ± 8.04) and in the INT (*M* = 2.02 ± 3.93) conditions, *t*_(12)_ = 0.92, *p* = 0.38. Channels with a rejection rate superior to 20% across the trials and trials with more than 10 bad channels were rejected. For each participant and stimulus condition, the remaining trials (~80 per condition) were averaged in sync with the stimulus onset, digitally transformed to an average reference and corrected for a baseline over the 100-ms pre-stimulus segment section.

### Data analyses

#### Number-conservation task

Prior to the data analyses, we discarded trials in which (a) the INT stimuli were presented before the COV stimuli (i.e., the distractor trials), (b) the number of tokens in the two rows differed, (c) artifacts, eye blinks or eye movements occurred, and (d) the participants committed errors or responded in less than 200 ms. Thus, we analyzed 78 ± 3 trials in the COV and 81 ± 2 trials in the INT conditions. After removing the outliers (1.5% of the trials), defined as RTs more than 2 *SD* from the mean of that participant, we averaged the RTs for each of the four different numbers of tokens in each condition (COV and INT). In addition, in the INT condition, the RTs were averaged for each increase in the distance between the tokens from the shorter to the longer row (i.e., 3, 5, or 7 mm).

Consistent with previous ERP studies (e.g., Whelan et al., 2010; Clayson and Larson, 2011a,b), the selection of the electrodes for analysis was made in regards to topographic distribution of the different components of interest (N2, P3, and late positive parietal components) and on the basis of ROIs in visuo-spatial tasks—i.e., the parieto-central electrodes for the N2 [see Jonkman (2006)]—and in the inhibition of the length-equals-number heuristic in children (Houdé et al., 2011; Poirel et al., 2012) and in adults (Daurignac et al., 2006; Leroux et al., 2006, 2009; Joliot et al., 2009)—i.e., the fronto-central electrodes for the P3 component. Figure 4 displays the ERP waveforms of each stimulus condition, COV and INT, at selected electrodes and the topography at the peak latency of the N2, P3, and late positive parietal components. Visual inspection of the ERP distribution showed a positive wave occurring over fronto-central sites from 300 ms, which we referred to as the P3. Over parietal sites, a negative component was recorded in the INT condition, peaking around 250 ms, whereas at the same time and location COV stimuli elicited a positive wave (hereafter referred as N2). Following this component, a late positive wave was recorded at parietal sites from 500 to 700 ms (i.e., late positive parietal component). Time windows of analysis have been chosen according to the topographic distributions of these components in the grand-average ERPs: 310–360 ms for the fronto-central P3, 200–350 ms for the centro-parietal N2 and 550–650 ms for the late positive parietal components. For the P3 component (see Figure 4), we chose a representative subset of 5 fronto-central channels over the left (LH: 29, 23, 16, 17, and 44) and right hemispheres (RH: channels 5, 6, 7, 198, and 185). For the N2 and late positive parietal components (see Figure 4), the representative subset of channels was composed of 5 parietal channels over each hemisphere (LH: 80, 89, 100, 110, and 118; RH: channels 127, 128, 129, 130, and 131). The maximum amplitude and latency were averaged for the 5 electrodes over the LH and also over the RH for the P3 and late positive parietal components. Because of the large differences between the two conditions for the N2 peak component, mean amplitudes were used in this case and were averaged for the electrodes over each hemisphere.

#### Mental imagery task

Altogether, 0.9% of the trials were deleted because the participants pressed the space bar in less than 200 ms and therefore the RTs could not reflect the process of interest, i.e., mental shortening of the row. Outliers defined by RTs more than 2 *SD* from the mean for that participant were also deleted. Outliers occurred in 1.3% of trials. RTs were then averaged for each increase in the distance between the tokens from the shorter to longer row (i.e., 3, 5, or 7 mm).

## Results

We initially ran a Condition (COV vs. INT) × Number of tokens (5 vs. 6 vs. 7 vs. 8) repeated measures analysis of variance (ANOVA) on the RTs in the number-conservation task to determine whether the participants counted the number of tokens in the two rows to perform the task. We then analyzed the RTs in the INT condition for the number-conservation and imagery tasks using a Task (number-conservation vs. mental imagery) × Distance difference between the tokens in the two rows (3 vs. 5 vs. 7 mm) repeated measures ANOVA to determine whether the RTs increased linearly with the distance by which the longer row had to be shortened to align with the tokens in a one-to-one correspondence. The ERP data for each component were analyzed within Condition (COV vs. INT) × Hemisphere (LH vs. RH) repeated measures ANOVA. Finally, we analyzed the correlations between (a) the slopes of the best-fitting lines in the INT condition of the number-conservation task and the mental imagery task, (b) the difference in the RTs and amplitudes of the ERP components between the COV and INT conditions, and (c) the slopes of the best-fitting lines in the INT condition of the number-conservation task and late positive parietal ERP components. For each of the analyses, we reported the effect size of the ANOVA (partial eta squared) or the difference of the means (Cohen's d).

### RTs

The Two-Way ANOVA revealed a typical interference effect: The participants determined faster that the two rows had an identical number of tokens in the COV (*M*_COV_ = 1204 ± 391 ms) compared to the INT (*M*_INT_ = 1396 ± 578 ms) condition, *F*_(1, 12)_ = 7.14, *p* < 0.05, η^2^_*p*_ = 0.37. There was a main effect of the number of tokens presented in the rows, *F*_(3, 36)_ = 8.10, *p* < 0.0001, η^2^_*p*_ = 0.40, but this effect varied in function of the condition, as witnessed by a significant interaction, *F*_(3, 36)_ = 5.56, *p* < 0.005, η^2^_*p*_ = 0.32. Critically, the cubic trend was significant in the INT condition, *F*_(1, 12)_ = 10.43, *p* < 0.01, η^2^_*p*_ = 0.47, which argued against the fact that participants counted the tokens in the two rows to determine whether the rows had the same number of tokens after one row was transformed in length (see Table 1a). Indeed, if one counts the tokens in each row to determine whether they possess the same number of tokens, then each additional token should take an extra time to be counted and therefore the linear but not the cubic trend should be significant.

**Table 1a:** **The mean response times and standard deviations in the number-conservation task for the four different numbers of tokens presented in the condition in which the number and length co-varied (COV) and in the condition in which the number and length interfered (INT)**.

	**COV condition**					**INT condition**				
	**Number of tokens**				**Total**	**Number of tokens**				**Total**
	**5**	**6**	**7**	**8**		**5**	**6**	**7**	**8**	
*M*	1127	1134	1277	1277	1204	1185	1284	1550	1567	1396
*SD*	306	309	466	468	391	361	482	690	678	578

**Figure 3:**
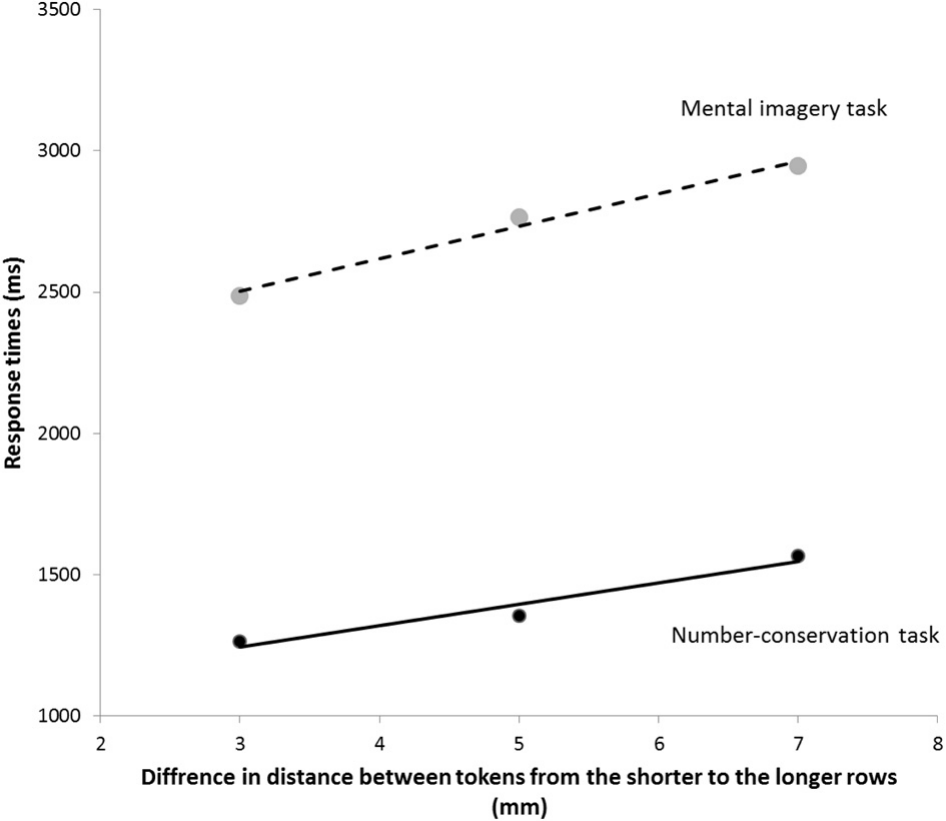
**Mean response times for the three differences in distance between the tokens in the two rows in the INT condition of the number-conservation task and the mental imagery task**. Solid lines denote the best-fitting lines for the RTs in the INT condition of the number-conservation task (plain) and the best-fitting lines for the RTs in the mental imagery task (dashed).

A Two-Way ANOVA for the RTs in the INT condition of the number-conservation task and in the mental imagery task revealed a main task effect with shorter RTs in the number-conservation task (*M* = 1396 ± 578 ms) than in the mental imagery task (*M* = 2733 ± 679 ms), *F*_(1, 12)_ = 28.38, *p* < 0.0001, η^2^_*p*_ = 0.71, a main effect of the distance between the tokens in the longer row, *F*_(1, 12)_ = 39.39, *p* < 0.0001, η^2^_*p*_ = 0.77, and a significant interaction with effects of the distance between the tokens that differed in the two tasks, *F*_(2, 24)_ = 5.28, *p* < 0.025, η^2^_*p*_ = 0.31. Critically, in both tasks, the RTs increased linearly with increasing distance between the tokens, as demonstrated by significant linear trends in the number-conservation task, *F*_(1, 12)_ = 72.37, *p* < 0.0001, η^2^_*p*_ = 0.86, and in the mental imagery task, *F*_(1, 12)_ = 30.89, *p* < 0.0001, η^2^_*p*_ = 0.72 (see Figure 3 and Table 1b). This result suggests that participants imagined the tokens in the longer row aligning with the tokens in the shorter row both in the mental imagery and in the number-conservation tasks. A comparison of the steepness of the slopes of the best-fitting lines revealed that participants imagined the shortening of the longer row more quickly in the number-conservation task (*M* = 75.9 ± 32.2 ms/mm) than in the mental imagery task (*M* = 114.3 ± 74.1 ms/mm), *t*_(12)_ = 2.16 *p* < 0.05, *d* = 0.71. Finally, the slopes of the best-fitting lines were significantly correlated in the two tasks, *r*_(11)_ = 0.51, *p* < 0.05, further suggesting that the same mental transformation process was at play in the mental imagery and in the number-conservation tasks. Given that we found no correlation between the overall RTs in the mental imagery task and the number conservation task, *r*_(11)_ = −0.05, *p* = 0.87, it is unlikely that the correlation between the slopes of the best-fitting lines are mediated by the difficulty of the tasks or general processing speed.

**Table 1b:** **The mean response times, mean slopes of the best-fitting line and standard deviations for the three differences in distance between the tokens in the two rows in the INT condition of the number-conservation and mental imagery task**.

	**Number-conservation task (INT condition)**					**Mental imagery task**				
	****									
	**Distance between tokens**			**Slope**	**Total**	**Distance between tokens**			**Slope**	**Total**
	**3**	**5**	**7**			**3**	**5**	**7**		
*M*	1264	1355	1568	75.9	1396	2488	2766	2946	114.3	2733
*SD*	509	588	548	32.1	578	597	699	767	74.1	679

### ERPs

#### Centro-parietal N2

**Figure 4:**
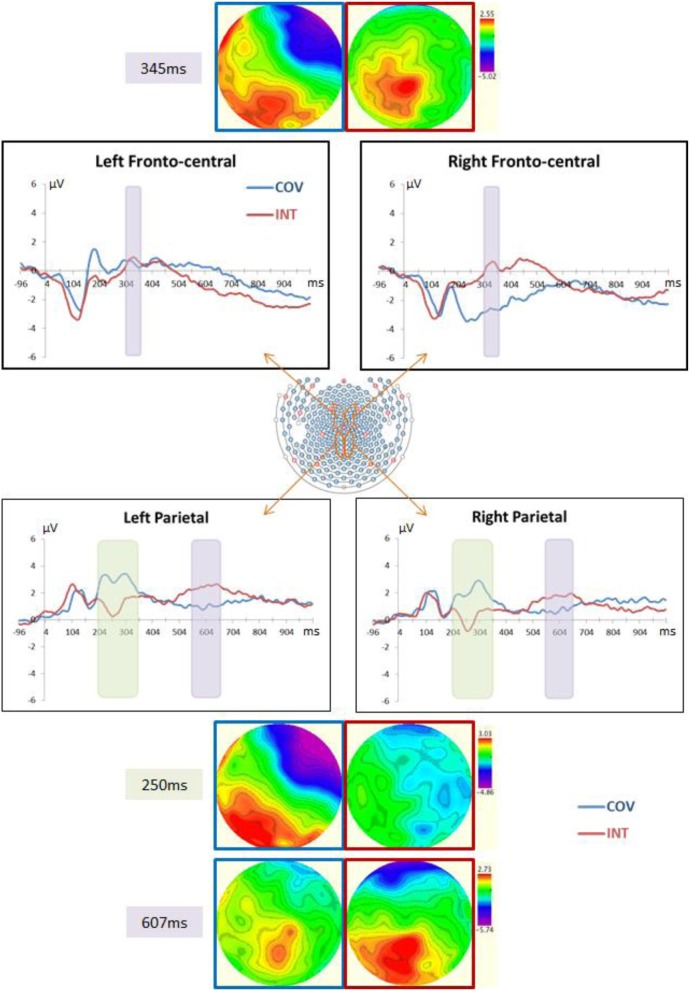
**Central panel: Depiction of the fronto- and parieto-central channel subsets used for the ERP analyses**. ERP waveforms in this figure correspond to the average of each channel subset depicted in orange. Top left and right panels: ERPs at, respectively, the left and right fronto-central channels in the COV (i.e., the two rows had the same length and number of tokens) and the INT (i.e., the two rows had different lengths but the same number of tokens) conditions. Waveforms and top view topographical maps (blue frame for the COV condition and red frame for the INT condition) show positive fronto-central potentials within 310–360 ms, especially in the INT condition. Bottom left and right panels: ERPs at, respectively, the left and right parieto-central channels in the COV and the INT conditions. Waveforms and top view topographical maps (blue frame for the COV condition and red frame for the INT condition) display negative centro-parietal potentials in the INT condition and positive potentials in the COV condition around 250 ms, followed by positive potentials larger in INT than in COV within 500–700 ms. Note that because the EGI system is not based on the standard 10/20 system referential, we did not refer to the standard 10/20 system to indicate the electrode positions.

The mean amplitude of the N2 component differed between the two hemispheres, *F*_(1, 12)_ = 15.43, *p* < 0.01, η^2^_*p*_ = 0.56, and was greater in the INT than COV condition, *F*_(1, 12)_ = 13.62, *p* < 0.01, η^2^_*p*_ = 0.53 (see Figure 4). We observed no correlation between the participants' efficiency to resist (inhibit) the length-number interference, as was measured by the difference in the RTs between the INT and COV conditions and the difference in the amplitude of this component between the two conditions in the LH, *r*_(11)_ = 0.38, *p* = 0.22, and RH, *r*_(11)_ = 0.44, *p* = 0.12. In addition, the amplitudes of the centro-parietal N2 component in the INT condition were not correlated with the increases in RTs with increasing number of tokens presented in the two rows (as reflected by the steepness of the slope of the best-fitting lines), *r*_(11)_ = −0.07, *p* = 0.82 in the LH and *r*_(11)_ = −0.10, *p* = 0.75 in the RH, or with the increases in RTs with increasing distance between tokens, *r*_(11)_ = −0.18, *p* = 0.56 in the LH and *r*_(11)_ = −0.15, *p* = 0.63 in the RH.

#### Fronto-central P3

The Two-Way ANOVA of the maximum amplitudes revealed a main effect of condition with a greater amplitude in the fronto-central P3 component in the INT than COV condition, *F*_(1, 12)_ = 7.21, *p* < 0.025, η^2^_*p*_ = 0.38, and a main effect of hemisphere, *F*_(1, 12)_ = 4.81, *p* < 0.05, η^2^_*p*_ = 0.29. Critically, the effect of the condition varied as a function of the hemisphere, *F*_(1, 12)_ = 5.00, *p* < 0.05, η^2^_*p*_ = 0.30: The larger amplitude of the fronto-central P3 for the INT condition was observed in the RH, *t*_(12)_ = 3.33, *p* < 0.005, *d* = 0.87, but not in the LH, *t* < 1 (see Figure 4). Finally, the participants' efficiency to resist (inhibit) the length-equals-number interference, as measured by the difference in RTs between the INT and COV conditions, was correlated with a difference in the amplitude of the fronto-central P3 component between the two conditions in the RH, *r*_(11)_ = 0.60, *p* < 0.05, but not the LH, *r*_(11)_ = −12, *p* = 0.69. Critically, we found no correlation between the amplitudes of the fronto-central P3 component in the INT condition and the increases in RTs with increasing number of tokens presented in the two rows, *r*_(11)_ = −0.11, *p* = 0.72 in the LH and *r*_(11)_ = −0.10, *p* = 0.75 in the RH, or between the amplitudes of this component and the increases in RTs with increasing distance between the tokens, *r*_(11)_ = 0.23, *p* = 0.45 in the LH and *r*_(11)_ = 0.32, *p* = 0.29 in the RH. Thus, the correlation observed between the difference in RTs between the INT and the COV conditions and the amplitude of the fronto-central P3 component is unlikely to be a byproduct of a confound between the variable manipulated (i.e., condition) and the difficulty of the task.

#### Late positive parietal

We observed a marginal effect of the hemisphere, *F*_(1, 12)_ = 4.61, *p* = 0.053, η^2^_*p*_ = 0.28, and condition, *F*_(1, 12)_ = 3.68, *p* = 0.08, η^2^_*p*_ = 0.24, on the amplitude of the late positive parietal component (see Figure 4). The amplitude of the late positive parietal component averaged over the two conditions tended to be higher in the LH (*M* = 1.76 ± 2.17 μV) than in the RH (*M* = 1.31 ± 2 μV). We observed no significant correlation between the slopes of the best-fitting lines in the INT condition of the number-conservation task and amplitude in the INT condition of the late positive parietal component in the LH, *r*_(11)_ = 0.31, *p* = 0.30, or RH, *r*_(11)_ = 0.23, *p* = 0.43. However, the speed at which the participants mentally shortened the longer row in the INT condition, reflected by the steepness of the slopes of the best-fitting lines, was correlated with the latency of this component in the LH, *r*_(11)_ = −0.56, *p* < 0.05, and RH, *r*_(11)_ = −0.55, *p* < 0.05, in the INT condition. Finally, to determine whether the correlation between the increases in RTs with increasing distance between the tokens and the latencies of the late positive parietal component could be due to the increasing difficulty to perform the task as the distance increases, we computed the correlation between the increases in RTs with increasing number of tokens presented in the rows and the latencies of the late positive parietal component. Indeed, as the number of tokens increases, the task becomes more difficult but this manipulation has no effect on the mental transformation processes possibly involved in that task. We found no correlation between the steepness of the slopes of the best-fitting lines computed on the number of tokens and the latencies of the late positive parietal component, *r*_(11)_ = −0.07, *p* = 0.82 in the LH and *r*_(11)_ = 0.22, *p* = 0.47 in the RH, which argues again the fact that difficulty in itself can explain the correlation reported between the latency of the late positive parietal component and the increases in RTs with increasing distances between the tokens.

## Discussion

Participants were faster to determine that two rows had an identical number of tokens in the condition in which the two rows had similar lengths (COV condition) than in the condition in which the two rows had different lengths (INT condition)—i.e., after the tokens in one of the row of the preceding COV stimulus were spread apart. This procedure mirrored, in that respect, the procedure used in Piaget's seminal number-conservation task (Piaget, 1952). The ERP analyses revealed that the N2 and P3 component amplitudes increased in the INT condition. These two ERP components are traditionally interpreted as indexing cognitive control processes, including conflict monitoring and inhibitory control (Nieuwenhuis et al., 2003; Anokhin et al., 2004). The effects of the condition (COV vs. INT) on the amplitudes of the N2 and P3 components replicates those reported in previous ERP studies of adults that used static COV and INT stimuli in an adaptation of Piaget's number-conservation task (Daurignac et al., 2006; Leroux et al., 2006, 2009; Joliot et al., 2009). Critically, we report for the first time that inhibitory control efficiency, reflected by the difference in the RTs between the COV and INT conditions, was related to the difference in the amplitudes of the frontal P3 components between the COV and INT conditions. The rightward lateralization of the frontal P3 component distribution is consistent with the activation of the right prefrontal cortex (i.e., the right inferior frontal gyrus/insula) reported in an fMRI study in children performing the number-conservation task (Houdé et al., 2011; Poirel et al., 2012). Together, the behavioral and neurophysiological data strongly suggest that adults must inhibit the length-equals-number heuristic to determine that a row of tokens that were spread apart has the identical number of tokens as a row that was not transformed in length during Piaget's number conservation task. We note that N2 is usually recorded with maximum amplitudes over fronto-central areas especially in tasks involving inhibition. However, as suggested by Folstein and Van Petten (2008), in some studies N2 difference wave could be much more prominent over posterior than anterior electrodes, especially in the visual modality. For instance, in an oddball task, Whelan et al. (2010) generated regions of interest according to the morphological characteristics of the ERPs and identified midline centro-parietal groups of electrodes for the N2 wave that followed target stimuli. Note that this study used the international 10/20 system but the electrodes listed are very close to the recording positions we selected for the N2 component in our study. Thus, the centro-parietal location of the N2 component most likely reflects that the inhibition of a heuristic highly reinforced in our visual environment requires an effortful control and therefore recruits additional attention areas in the parietal cortex (Jonkman, 2006).

One could argue that the N2 and P3 components are unlikely to reflect inhibitory control processes given that the apparent movement lasted for 500 ms and therefore inhibition should occur after the apparent movement has stopped. However, we want to argue that inhibition of the length-equals-number heuristic might actually be triggered as soon as an interference occurs between the lengths of the rows and the number of tokens in that rows. In the INT stimulus, this interference emerged as soon as the movement started. Indeed, all ERPs analyses presented in the present study were restricted to trials in which a COV stimulus with identical number of tokens in the two rows preceded the INT stimulus. Critically, no tokens were added or taken away in the two rows between the COV and the INT stimuli. Thus, the critical difference between the COV and the INT stimuli regarded the co-variance (or interference) between the lengths of the rows and the number of tokens and this difference emerged as soon as the apparent movement started. In the COV stimuli, the apparent movement was such that at any given time of this movement the longer row contained more tokens that the shorter rows (each token appearing one after the other) until both rows had the same length and the same number of tokens. Conversely, in the INT stimuli, as soon as the apparent movement started (i.e., lengthening of one of the row of tokens) the length of the rows interfered with the number of tokens given that both rows had the same length and the same number of tokens at the onset and in order to resist to this interference participants needed to inhibit the length-equals-number strategy. Therefore, the difference in the amplitudes of the N2 and P3 components might well reflect inhibitory control processes.

One could also argue that the topographic maps revealed distribution of the N2 and P3 components that are not consistent with the distribution of these components in classical inhibitory tasks (such as inhibition of a motor response, e.g., go/no-go tasks) and thus that these components do not reflect inhibition processes. The distribution of these components and particularly the difference in the distributions between the COV and the INT conditions might be subtle because as opposed to classical inhibitory control tasks, the number-conservation tasks was originally designed by Piaget (1952) to evaluate children's conceptual knowledge of number. Therefore, the number-conservation task might rely on inhibition processes that are largely automatized in adults: Adults as opposed to children have encountered number of situations in which length and number interfered—situations in which the length-equals-number heuristic must be inhibited. The repetitive inhibition of this heuristic might have contributed to automatize this inhibition process. This might be especially true in a context in which the interference emerged after length and number covaries as in Piaget's classical number-conservation task. This hypothesis is consistent with the fact that the modulation of the fronto-central P3 components occurred during the apparent movement—i.e., before one could judge the numerical equivalence of the two rows. Finally, we note that a hallmark of the resistance to interference and of inhibitory control at a behavioral level—i.e., the difference in RTs between the COV and the INT stimuli—was correlated with the difference in the amplitude of the fronto-central P3 between the COV and the INT conditions. In addition, this difference was maximal over the right fronto-central channels—a topography consistent with previous fMRI findings revealing that the acquisition of number conservation relies on the activation of the right inferior frontal gyrus (Houdé et al., 2011; Poirel et al., 2012)—an area critically involved in resisting interference (Wager et al., 2005) and in the inhibition of a dominant response (Aron et al., 2004). Therefore, in light of previous results reported on the number-conservation task on children (Houdé and Guichart, 2001; Houdé et al., 2011; Poirel et al., 2012) and adults (Daurignac et al., 2006; Leroux et al., 2006, 2009; Joliot et al., 2009), it is not unlikely that the difference in the amplitude of the P3 components at the fronto-central channels between the COV and the INT conditions reported in the present study reflected the involvement of executive control processes. That said, we note that a limitation of the present study was that apriori ROIs were used to reveal that adults still needed to inhibit the length-equals-number heuristic to perform Piaget's number-conservation task. This procedure was necessary given that adults might have largely automatized the inhibition of the length-equals-number heuristic especially in a context such as the number-conservation task.

Finally, the difference in the N2 and P3 amplitudes between the INT and the COV conditions might simply reflect difference in the difficulty, attention or extent of eye movements between these two conditions. Although difficulty was greater in the INT than the COV conditions, we note that this is the case for all paradigms assessing inhibitory control efficiency (e.g., Stroop task, Flanker task, Simon task). The critical question is to determine why the incongruent condition is more difficult than the congruent one and what additional processes are needed to perform the incongruent condition relative to the congruent one. For instance, in the Stroop Color-Word task (Stroop, 1935), difficulty increases in the incongruent condition because one needs to inhibit reading the word denoting a color in order to identify the color of the ink the word is printed in. Similarly, in the number-conservation task, we want to argue that the difficulty of the incongruent condition is driven mainly by the need to inhibit an overlearned strategy or heuristic (i.e., length-equals-number). Except for the critical manipulation of the covariance between the length of the rows and the number of tokens, all other aspects of the two conditions (apparent movements on the screen, number of tokens, visual angle) were equated in the two conditions. In addition, the number of segments rejected due to eye movements was identical in the COV and the INT conditions. Therefore, we are confident that the difference in behavioral performance and ERP amplitudes between the COV and the INT conditions reflected at the very least the ability to resist to interference, here the interference between length and number. In addition, one could also argue that the differences in amplitude of the centro-parietal N2 and fronto-central P3 components observed between the COV and the INT condition as well as the laterality effect reported might be due to a contamination from motor-related potentials. However, we note that all participants provided responses with their right hand and that response occurred more than 1200 ms after stimulus onset well beyond the time-windows from which the amplitudes of the components were extracted.

When the length-equals-number heuristic is successfully inhibited, one must activate the appropriate strategy to determine whether two rows of different lengths have an identical number of objects. Previous studies have suggested that a counting strategy might be used (Daurignac et al., 2006; Leroux et al., 2006, 2009; Joliot et al., 2009). However, the presentation of the transformation of one of the physical dimensions of the display (e.g., the length of the row) without an alteration of the other physical dimensions (e.g., the number of tokens) allows one to succeed in the number-conservation task by grasping the principle of the reversibility of operations, i.e., the movement from A to B can be nullified by the movement from B to A (Piaget, 1952; Elkind, 1967), without actually counting the number of objects in each row. Consistent with Piaget's view, the response times in the present study did not increase linearly with the number of tokens when the two rows were of different lengths, which argues against a counting strategy. However, the response times increased linearly with an increasing difference in the distance between the tokens from the shorter to the longer rows. This effect is consistently interpreted as showing that mental images are transformed in a manner that emulates the corresponding physical transformation (e.g., Kosslyn et al., 1978, 2006; Borst and Kosslyn, 2008; Moulton and Kosslyn, 2009). Thus, the linear increase of the response times with an increasing transformation of the physical dimension is consistent with the hypothesis that, after the transformation of the length of one row, the participants mentally shortened the longer row to align the tokens in the two rows in a one-to-one correspondence to determine that both rows have an identical number of tokens. This result is further supported by the relationship observed between the slopes of the best-fitting lines in the number-conservation task and those in the mental imagery task, in which the participants were explicitly asked to mentally shorten the row of tokens to align them with ones in another row in a one-to-one correspondence. The ERP analyses revealed that a late positive parietal component was affected by the interference between the length of the rows and number of tokens (i.e., the INT condition). This late positive parietal component might reflect the process of visualizing the transformation of the longer row (i.e., a shortening of the row), as suggested by previous ERP studies on mental rotation that reported a modulation of the amplitude of a late positive parietal component by the angular disparity between objects, i.e., the amplitude of the late positive parietal component decreased with increasing angular disparity between the objects (e.g., Heil, 2002; Schendan and Lucia, 2009; Beste et al., 2010; Ter Horst et al., 2012). Finally, participants who could mentally shorten the longer row more quickly were the ones that had a greater latency of the late positive parietal component in the ERP analysis, which further indicates that this late positive parietal component could index mental transformation processes in the number-conservation task. The involvement of the parietal cortex in the reversibility of operations in the number-conservation task is consistent with the relationship between the activation of the intraparietal sulcus and ability to manipulate information in working memory, particularly to repeat number sequences in reverse order (Poirel et al., 2012), that was reported in a previous fMRI study on children performing the number-conservation task.

A limitation of the present study is that we only provided indirect evidence that mental transformation processes were at play in the number-conservation task. To provide direct evidence that participants imagine the shortening of the row that was transformed in length, one needs to demonstrate that the difference in the distance between the tokens from the shorter to the longer rows affects the amplitude of the late positive parietal component (e.g., Heil, 2002; Schendan and Lucia, 2009; Beste et al., 2010; Ter Horst et al., 2012). This was not possible in the present study due to the limited number of trials presented in each increase in the distance between the tokens from the shorter to the longer row (i.e., 3, 5, or 7 mm). In addition, the late positive parietal component does not only reflect mental transformation processes. Indeed, late positive parietal components (such as the P3b) index multiple cognitive processes including encoding and rehearsal of the working memory processes (Joliot et al., 2009) or the violation of arithmetic rules (Núñez-Peña and Honrubia-Serrano, 2004). That said, several visual mental imagery studies reported that the amplitude of the late positive component that occurs between 300 and 700 ms after the stimulus onset is larger in the imagery than in the control condition (Wu et al., 2006; Schendan and Ganis, 2012). In the present study, given that the ability to mentally transform an image (as reflected by the steepness of the slope of the best-fitting line computed on the increase in distance between the tokens from the longer to the shorter rows) but not the ability to count tokens (as reflected by the steepness of the slope of the best-fitting line computed on the increase in the number of tokens) was correlated with this late positive parietal component, it is likely that this component reflected mental transformation processes.

Considering behavioral and neurophysiological data, we wish to argue that to succeed at Piaget's number-conservation task, one must initially inhibit the length-equals-number misleading strategy to activate a reversibility that imagines the shortening of the row that was lengthened. Because adults rely on imagining the shortening of the longer row (i.e., empirical reversibility) to perform the number-conservation task and children as young as 4 years old can manipulate mental images (Marmor, 1975, 1977; Kosslyn et al., 1990), our data collected on adults suggest that the acquisition of number conservation could be partially rooted in the ability to emulate the reverse physical transformation that just occurred using mental imagery. However, to activate such visuo-spatial reversibility, one must be able to inhibit the length-equals-number misleading strategy, which could explain why a previous study failed to observe a relationship between number conservation and mental rotation (Marmor, 1977).

In conclusion, the data collected in the present study suggest that number conservation relies on two critical mechanisms in adults: inhibitory control and the reversibility of operations. Initially, one must inhibit a misleading heuristic to then activate the reversibility of operations, i.e., imagining a shortening of the row that was lengthened. Observing that adults must inhibit the length-equals-number heuristic suggests that we never fully outgrow the cognitive and perceptual biases of our childhood (Diamond and Kirkham, 2005; Borst et al., 2013). Finally, observing that mental imagery supports the reversibility of operations and acquisition of number conservation, using a high-density ERP, further questions seminal psychological models, in which the outcome of cognitive development is a system rooted in formal operations (Piaget, 2001) or language (Bruner, 1964). Currently, we must reconsider the exploration of the role of mental imagery, coupled with executive functions, in cognitive development.

### Conflict of interest statement

The authors declare that the research was conducted in the absence of any commercial or financial relationships that could be construed as a potential conflict of interest.
